# Mechanism of Yellowing: Carbonyl Formation during Hygrothermal Aging in a Common Amine Epoxy

**DOI:** 10.3390/polym10091017

**Published:** 2018-09-13

**Authors:** Andrey E. Krauklis, Andreas T. Echtermeyer

**Affiliations:** Department of Mechanical and Industrial Engineering, Norwegian University of Science and Technology, 7491 Trondheim, Norway; andreas.echtermeyer@ntnu.no

**Keywords:** epoxy, yellowing, mechanism, thermo-oxidation, carbonyl formation, leaching

## Abstract

Epoxies are often exposed to water due to rain and humid air environments. Epoxy yellows during its service time under these conditions, even when protected from UV radiation. The material’s color is not regained upon redrying, indicating irreversible aging mechanisms. Understanding what causes a discoloration is of importance for applications where the visual aspect of the material is significant. In this work, irreversible aging mechanisms and the cause of yellowing were identified. Experiments were performed using a combination of FT-NIR, ATR-FT-IR, EDX, HR-ICP-MS, pH measurements, optical microscopy, SEM, and DMTA. Such extensive material characterization and structured logic of investigation, provided the necessary evidence to investigate the long-term changes. No chain scission (hydrolysis or oxidation-induced) was present in the studied common DGEBA/HDDGE/IPDA/POPA epoxy, whilst it was found that thermo-oxidation and leaching occurred. Thermo-oxidation involved evolution of carbonyl groups in the polymeric carbon–carbon backbone, via nucleophilic radical attack and minor crosslinking of the HDDGE segments. Four probable reactive sites were identified, and respective reactions were proposed. Compounds involved in leaching were identified to be epichlorohydrin and inorganic impurities but were found to be unrelated to yellowing. Carbonyl formation in the epoxy backbone due to thermo-oxidation was the cause for the yellowing of the material.

## 1. Introduction

Epoxy resins are used worldwide as adhesives, as matrices in composites, as surface coatings, as casting materials, and as laminating agents for artwork, longboards, and guitars [1,2,3,4]. Epoxies are also used extensively for glass conservation [5,6], as adhesives and gap-fillers [7,8], and in preservation of outdoor architectural and monumental stone as adhesives and as injection grouts for filling cracks, as well as consolidants for porous, fragile deteriorated stone [9]. In most of these applications, stability of color is of importance.

Epoxies are two-component systems consisting of an epoxy compound and a hardener, which react to form the cured network [8]. In this study, the network of interest contains two epoxy compounds (DGEBA and HDDGE) and two hardeners (IPDA and POPA). After reaction, a three-dimensional amine epoxy network is obtained [10]. Epoxy resin family covers a large diversity of polymer networks by the type of the epoxy compound and hardener employed. All these variations lead to a different final network formed, and affect its physical and chemical properties [8,11]. In addition, commercial products often contain such components as plasticizers, diluents, accelerators, and trace impurities, which can affect the final properties and the yellowing [8,9]. Whilst DGEBA constitutes more than 75% of the market [12], the number of articles concerning aging of this particular resin (DGEBA/HDDGE/IPDA/POPA) is very limited [13,14]. Furthermore, yellowing is not investigated in any of them.

Epoxy resins are often exposed to water due to rain, humidity of the air, and in subsea and offshore applications [13,15]. In such conditions, water molecules can migrate into the polymer and may affect its properties, and lead to leaching [1,16]. Epoxies tend to yellow even at room temperature, even at medium humidity levels, with or without light exposure; it is a common observation [5,6,9]. However, light exposure can cause yellowing too, due to photo-yellowing (via photo-oxidation mechanism) [5,6,8,9,17]. In one study, it was observed that when exposed to high temperatures epoxy does not change its color in vacuum, while it does in air [18]. The yellowing phenomenon has often precluded wider use of epoxies in the various aforementioned applications [5,9]. However, studies on the yellowing of epoxy resins are few [5]. Furthermore, the identification of the mechanism of epoxy yellowing would be valuable in choosing suitable conditions, compounds or additives, for increasing service life of epoxies, i.e., in conservation [5,6].

Experimental evidence in this work was obtained using a combination of FT-NIR, ATR-FT-IR, EDX, HR-ICP-MS, pH measurements, optical microscopy, SEM, and DMTA. Such extensive material characterization, following the structured logic of investigation presented in this work, was novel and provided the necessary evidence to investigate the long-term changes in chemical structure of the studied resin.

The aim of this paper is to investigate the mechanistic origin of yellowing in a commonly used DGEBA/HDDGE/IPDA/POPA amine epoxy resin.

## 2. Theory

### 2.1. Irreversible Degradation Mechanisms

The hygrothermal process may involve both reversible and irreversible processes [19,20]. Irreversible changes persist even after redrying the material [13]. The resin yellows irreversibly, indicating that the mechanistic origin of the color change lies among irreversible degradation pathways.

The phenomenon of epoxy yellowing has been attributed to the photo-degradation of the amine hardener, to the degradation of the amine epoxy network itself via various pathways, to the degradation of additives or accelerators, and to the presence of impurities [9]. Degradation of the amine epoxy network may follow such pathways as chain scission, crosslinking between segments, and thermal and photo-oxidation of the main chains or sidegroups [18,21].

Irreversible aging mechanisms, which have been reported in the literature to occur during hygrothermal influence on general epoxies are [13,20,22,23]:

1. Hydrolysis (involves chain scission)
2. Thermo-oxidation (might involve chain scission, backbone modifications or crosslinking)
3. Photo-oxidation (might involve chain scission, backbone modifications or crosslinking)
4. Residual curing (additional crosslinking)
5. Leaching (initially present additives, impurities or degradation products)

In this work, photo-oxidative effects were avoided by conducting experiments in the absence of high-intensity light sources [5,6]. For the studied material, no hydrolysis occurred [14]. To avoid residual curing, the material was fully cured as was indicated by the total disappearance of exothermal signal via differential scanning calorimetry (DSC) [4,24]. Two runs were performed: The first run was at 80 °C for 16 h, and the second heating cycle was performed at 80 °C for 1 h, showing that no further hardening occurs. Based on this, hydrolysis, photo-oxidation, and residual curing were excluded, whilst thermo-oxidation and leaching were investigated further, in respect to the yellowing.

### 2.2. Thermo-Oxidation and Leaching

The chemical mechanism of epoxy thermo-oxidation is convoluted, and the exact degradation chemistry remains the subject of ongoing work [25]. The process is complex as it involves oxygen diffusion and consumption, and a radical chain mechanism initiated by hydroperoxide decomposition [26]. The process seems to obey Arrhenius law, with activation energies around 60–80 kJ/mol, for various epoxies [25,26]. Thermo-oxidation may proceed differently for epoxies with different structures and flexibility of the chains [27].

Thermo-oxidation of amine epoxies follows a general autoxidation mechanism, in which the main source of radicals is the decomposition of hydroperoxides [4,25]. Processes often involved during oxidation are chain scission, carbonyl formation, double bond formation, and amide formation [4,11,23,28,29].

There is a limited number of articles regarding epoxy leaching [30,31,32,33,34]. Moreover, leaching behavior of DGEBA/HDDGE/IPDA/POPA epoxy is not investigated in any of them. The leaching phenomenon may occur due to initially present additives, impurities, or degradation products diffusing out from the epoxy network into the water environment, which is in contact with the polymer. Often but not always, it follows Fickian type diffusion [30]. Commonly reported epoxy leaching compounds are bisphenol A or epichlorohydrin [30,31,32,33]. The driving force of this process is expected to be due to the difference in concentration of these chemicals inside the resin, and in the aqueous environment.

## 3. Materials and Methods

### 3.1. Materials

Amine-cured epoxy resin was prepared by mixing reagents Epikote Resin RIMR 135 (Hexion, Columbus, OH, USA) and Epikure Curing Agent MGS RIMH 137 (Hexion, Columbus, OH, USA) stoichiometrically, in a ratio of 100:30 by weight. The mixture was degassed in a vacuum chamber for 0.5 h to remove bubbles. The samples were cured at room temperature for 24 h, and post-cured in an air oven (Lehmkuhls Verksteder, Oslo, Norway) at 80 °C for 16 h. Full cure was achieved as described above in Reference [24]. The samples were cast into rectangular moulds and then cut into 40 × 7 × 2 mm^3^ rectangular samples with a vertical bandsaw. Sample preparation was followed by grinding with sandpaper (FEPA P60, grain size 269 µm). The sample geometry was chosen in accordance with standard practice for glass transition temperature determination, as described in Reference [35]. The dimensions were achieved within 5% tolerance.

**Figure 1:**
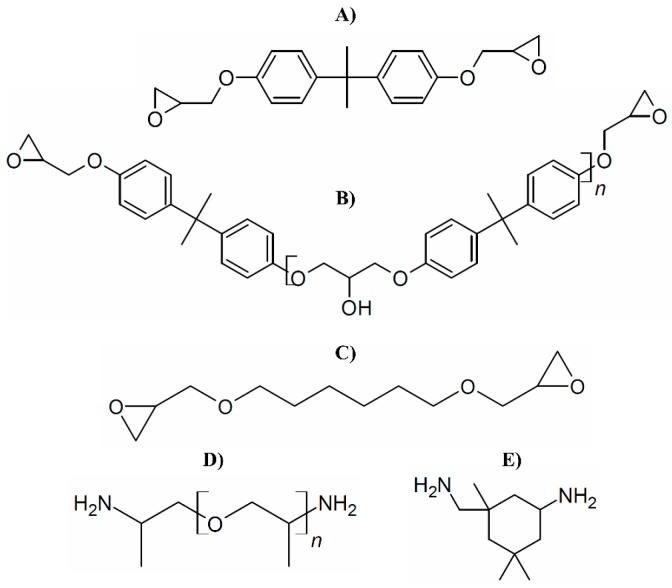
Molecular structures of epoxy and hardener components: (**A**) DGEBA monomer; (**B**) DGEBA oligomer (n = 1–2); (**C**) HDDGE; (**D**) POPA; (**E**) IPDA.

Resin and hardener (the epoxy system) consist of the following compounds: 0.63 wt % Bisphenol A diglycidyl ether (DGEBA; CAS 1675-54-3; number average molecular weight ≤ 700); 0.14 wt % 1,6-hexanediol diglycidyl ether (HDDGE; CAS 16096-31-4); 0.14 wt % poly(oxypropylene)diamine (POPA; CAS 9046-10-0; molecular weight 230); and 0.09 wt % isophorondiamine (IPDA; CAS 2855-13-2). Chemical structures of these compounds are shown in Figure 1.

The distilled water (resistivity 0.5–1.0 mΩ·cm) was used for conditioning, produced via the water purification system Aquatron A4000 (Cole-Parmer, Vernon Hills, IL, USA). pH of distilled water was determined to be 5.65, being slightly acidic as water is equilibrated with the atmospheric CO_2_.

### 3.2. Experimental Methods

#### 3.2.1. Overview

The hygrothermal conditions provide means to the accelerated aging, to study the effect of thermo-oxidation and leaching on the yellowing of epoxy. As was mentioned elsewhere in Reference [36], aging studies are intended to accelerate the degradation chemistry. The experimental evidence on the irreversible degradation mechanisms was obtained and reported in this work.

The analysis of yellowing and morphological characterization was performed using a combination of visual inspection, optical microscopy, and scanning electron microscopy (SEM, Tescan, Brno, Czech Republic). Chemical composition and macromolecular changes were studied using a combination of FT-NIR, ATR-FT-IR, EDX, HR-ICP-MS, pH measurements, and DMTA.

#### 3.2.2. Conditioning of Resin Samples in Distilled Water

Water uptake experiments were conducted using a batch system. A heated distilled water (60 ± 1 °C) bath was used for conditioning the samples. Samples were weighed using analytical scales AG204 (± 0.1 mg; Mettler Toledo, Columbus, OH, USA). Samples were conditioned for a period of two months. Samples were taken out of the water bath, weighed, and analyzed using a FT-NIR method [37].

#### 3.2.3. Drying of Conditioned Resin Samples

The drying of saturated samples was performed in a drying cabinet PK-410 (ESAB, London, UK), at 60 ± 1 °C in air atmosphere, with natural convection and relative humidity of 13 RH%. After that, samples were reconditioned at ambient conditions in the air to regain their initial water content.

#### 3.2.4. Optical Microscopy

Optical microscopy was performed using a digital microscope RH-2000 (Hirox, Tokyo, Japan), equipped with lens MXB-2500REZ, with a magnification of 140, and resolution of 1.06 µm.

#### 3.2.5. SEM and EDX

Scanning electron microscopy (SEM) and Energy-dispersive X-ray spectroscopy (EDX) experiments were performed using Mira/LMU (Tescan, Brno, Czech Republic) in backscattered electron regime, with working voltage of 15 kV.

#### 3.2.6. FT-NIR

Near-infrared (NIR) spectra were obtained using a Fourier transform spectrophotometer NIRSystems 6500 (Foss, Eden Prairie, MN, USA) operated in a transmission mode; an optical fiber probe and spectral analysis software Vision (Foss, Eden Prairie, MN, USA) was used. Spectra were taken in Vis-NIR wavenumber range of 4000–25,000 cm^−1^, using 32 scans per spectrum with a resolution of 4 cm^−1^.

FT-NIR spectroscopy was used to determine that the initial and redried epoxy has the same water content [37].

#### 3.2.7. ATR-FT-IR

Fourier transform infrared (FT-IR) spectra were recorded using Scimitar 800 FT-IR (Varian, Inc., Palo Alto, CA, USA) in the Attenuated Total Reflectance (ATR) mode via GladiATR^TM^ (Pike Technologies, Fitchburg, WI, USA). Spectra were obtained at 4 cm^−1^ resolution, co-adding 50 scans over a range of wavenumbers from 400 to 4000 cm^−1^.

#### 3.2.8. HR-ICP-MS

High resolution inductively coupled plasma mass spectrometry (HR-ICP-MS) analyses were performed using a double focusing magnetic sector field HR-ICP-MS Finnigan ELEMENT 2 (Thermo Fisher Scientific, International, Waltham, MA USA). A sample introduction system PrepFAST (ESI/Elemental Scientific, Omaha, NE, USA) was used. Pretreatment/digestion was done using UltraClave (Milestone, Milan, Italy). Acidification of samples was performed using ultra-pure grade HNO_3_ SubPur (Milestone, Milan, Italy), to avoid adsorption of ions to the wall of the vial.

#### 3.2.9. pH Measurements

pH measurements were performed using standard pH-meter MeterLab PHM210 (Radiometer analytical, Lyon, France) (pH ± 0.01). IUPAC standard buffer solutions (Radiometer analytical, Lyon, France) were used for calibration of pH meter.

#### 3.2.10. DMTA

Dynamic Mechanical Thermal Analysis (DMTA) tests, for determination of glass transition temperature, storage, and loss moduli were conducted using a Netzsch GABO qualimeter Eplexor, equipped with a 1.5 kN load cell (Netzsch GABO Instruments, Ahlden, Germany) operated in displacement control with a constant static strain of 0.4%, and a cyclic strain of 0.1% applied with a frequency of 1 Hz. The temperature sweep range was from 20 up to 120 °C, with a heating rate of 1 °C/min. The glass transition temperature (*T*_g_) was determined using DMTA as the crossing of tangents to the inflection points in the storage modulus curves [13,35].

## 4. Results

### 4.1. Discoloration and Changes in Morphology

**Figure 2:**
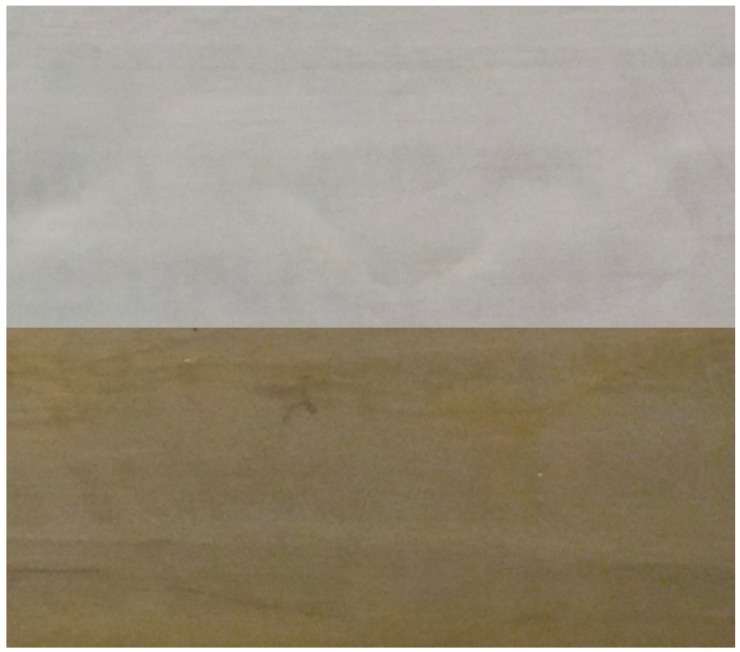
Visual inspection of an epoxy resin showing discoloration due to hygrothermal aging. **Top**: initial (blueish grey); **bottom**: redried after conditioning (yellow).

A difference in color of unaged versus aged epoxy is shown in Figure 2. The discoloration persists even after drying and reconditioning in air. Thus, the change in color was irreversible.

Yellowing was not a surface phenomenon, since change in color occurred homogeneously in the bulk, as was suggested by uniform color in the cross-section of the sample cut in the middle. This agreed with an observation made in another study [5].

**Figure 3:**
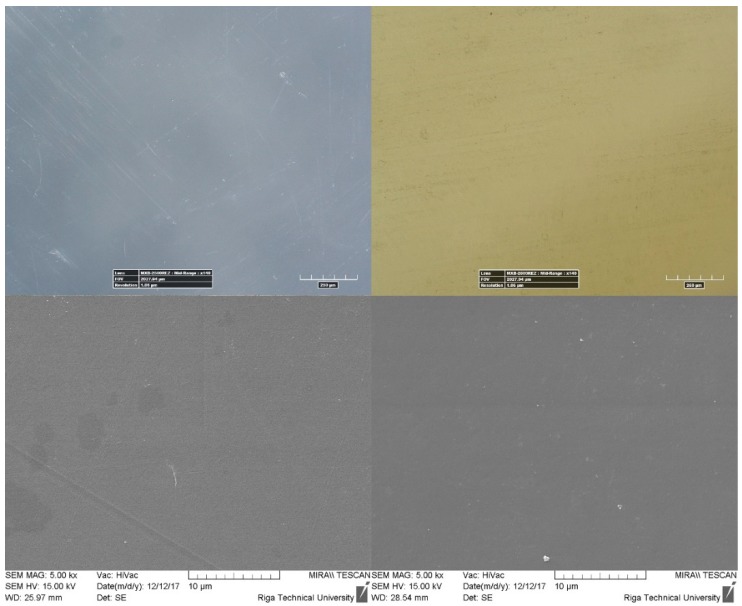
Digital optical (**top**) and SEM (**bottom**) micrographs of initial dry (**left**) and redried after hygrothermal aging (**right**).

Digital optical and SEM micrographs indicated that there was no observable change in morphology after hygrothermal aging and reconditioning to the initial water content, as shown in Figure 3. Surface morphology was studied for both aged and unaged material. It showed no significant changes. No pores or cavities were observed, suggesting no loss of polymer from the surface.

### 4.2. Changes in Chemical Composition

**Figure 4:**
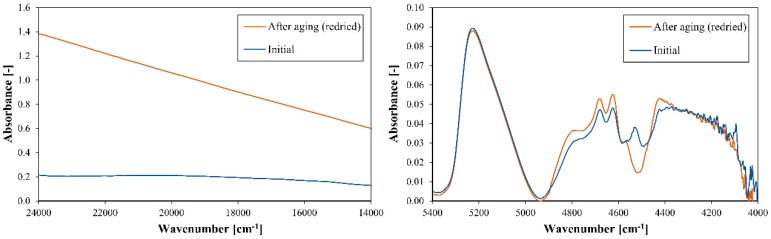
FT-NIR spectra of initial resin and redried epoxy after hygrothermal aging. (**Left**): visible light region. (**Right**): NIR region.

Obtained FT-NIR spectra of initial epoxy and resin redried to initial water content are shown in Figure 4.

Visible light region spectra indicated clearly, the yellowing and reduced transparency of the aged epoxy. In the NIR region of the spectra, both initial and redried epoxy after aging were similar with an exception of the peak at around 4535 cm^−1^, which corresponds to the epoxy ring [38]. It was observed that after hygrothermal aging, this peak had reduced dramatically, indicating that a compound, containing an epoxy ring, either leached or reacted. Since the resin was fully cured, the authors believe this peak corresponded to the leached unreacted epichlorohydrin, which contained an epoxy ring in its chemical structure. This claim was further supported with EDX and HR-ICP-MS.

**Figure 5:**
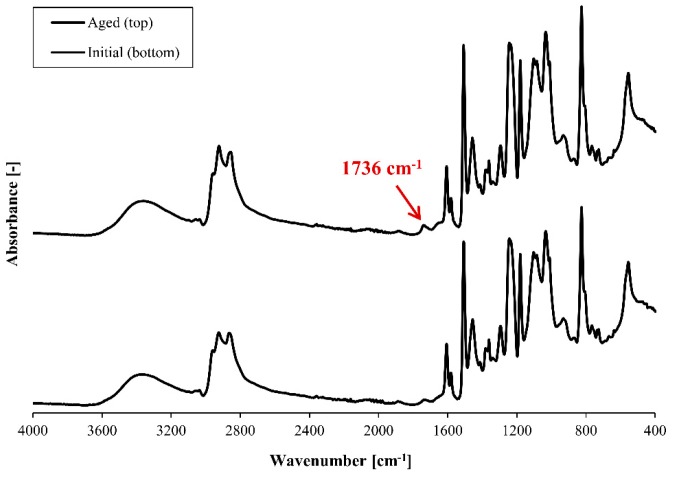
ATR-FT-IR spectra of initial (**bottom**) and redried after hygrothermal process resin (**top**).

Obtained ATR-FT-IR spectra of the initial dry epoxy and epoxy after hygrothermal process, drying and conditioning in air to initial water content, are shown in Figure 5.

Obtained spectra indicated that there was no significant difference observed in chemical structure of the initial and dried samples, except for the peak at 1736 cm^−1^, corresponding to carbonyl groups (νC=O) [4,8,10,28,39,40].

Elemental analysis via EDX indicated that initial dry epoxy had a higher content of Cl (0.99 ± 0.08 Cl%) than epoxy, after hygrothermal aging (0.73 ± 0.05 Cl%). This suggested leaching of chlorine-containing compounds from the resin into water during hygrothermal aging. This could be due to unreacted epichlorohydrin being released, which comes from the epoxy component. Moreover, oxygen content in the aged epoxy (23.59 ± 0.42 O%) was lower than in the initial one (24.35 ± 0.45 O%), indicating most likely a leaching of an epoxy ring-containing compound, such as unreacted epichlorohydrin.

**Figure 6:**
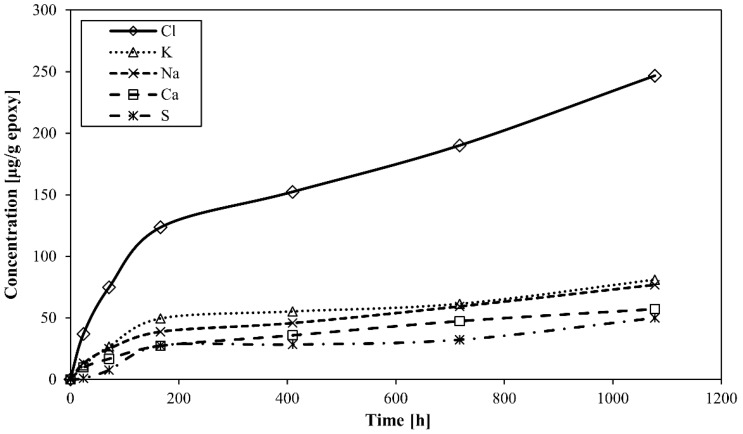
Ca, Cl, K, Na and S release from neat resin during hygrothermal aging at 60 °C.

Elements (Ca, K, Na, Cl, S) were identified to be leached by the resin into contacting water during hygrothermal aging using HR-ICP-MS. The element release curves are shown in Figure 6, with Cl release being dominant.

**Figure 7:**
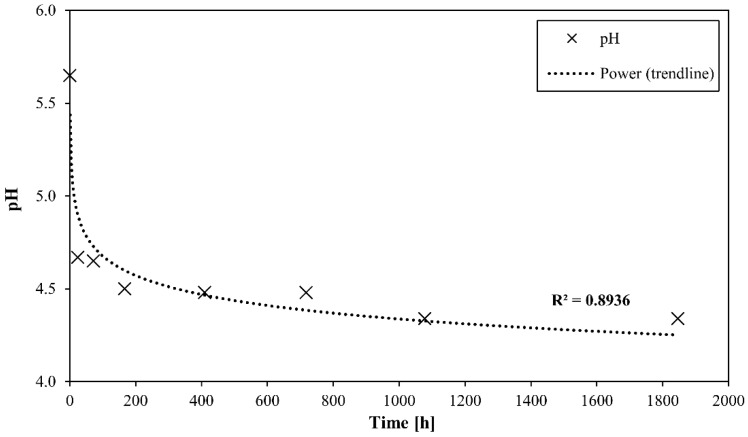
pH measurements of distilled water samples after contact with the resin.

pH measurement results of the water (50 mL samples) in contact with the resin during hygrothermal aging are shown in Figure 7. There was a strong initial decrease in pH upon contact of the dry resin with distilled water. The authors believe this could be related to a release of acidic impurities from the resin, i.e., HCl.

**Figure 8:**
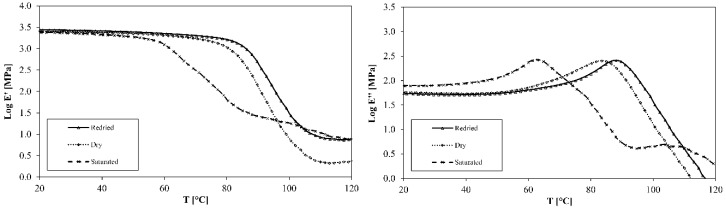
Temperature sweep for glass transition temperature determination for initial (dry); saturated; and redried. (**Left**): temperature sweep of tensile storage modulus. (**Right**): temperature sweep of tensile loss modulus.

The changes at macromolecular scale have been investigated by *T*_g_ change. The glass transition temperature (*T*_g_) of the initial, saturated, and redried epoxy was 81.7, 59.1, and 84.7 °C, respectively. *T*_g_ for saturated epoxy was much lower than for the dry and dried resin, due to plasticization [14]. The *T*_g_ of a redried epoxy was slightly higher than that of initial resin. In case of chain scission, a *T*_g_ value would have decreased [41], which was not observed. A slight increase in *T*_g_ of the dried material was likely due to polymer relaxation [13], minor thermo-oxidative crosslinking [7,27], or leaching of plasticizing compounds. The tensile storage and loss moduli in a temperature sweep range from 20 to 120 °C are presented in Figure 8.

## 5. Discussion

### 5.1. Logic of the Investigation

**Figure 9:**
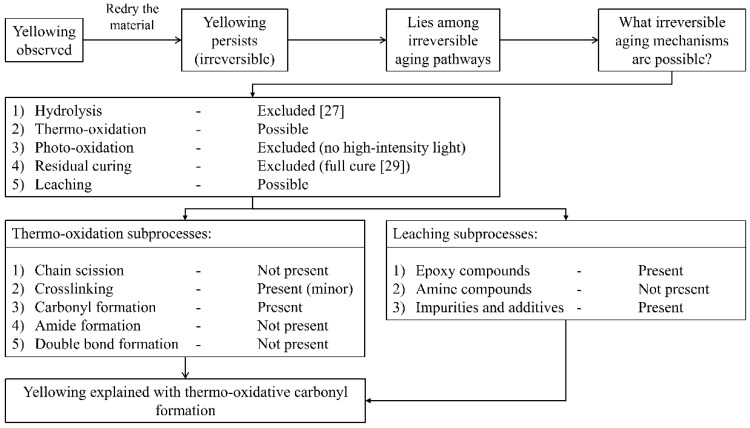
Schematic representation of the logic during investigation.

The logic which is followed in this study is shown schematically in Figure 9.

### 5.2. Leaching

There are three types of leaching that are potentially possible: (1) Leaching of hardener; (2) leaching of epoxy compounds; and (3) leaching of impurities or additives.

In theory, it is possible that some amount of unreacted hardener (blue in color), since soluble in water, would be washed out from crosslinked polymer network, or would be used up in additional crosslinking. The initial uncured epoxy resin is yellowish in color, which could explain the yellowing of the product over time [42]. The residual crosslinking, can also cause the decrease in unreacted amine group concentration causing the change in color [13], but this was not the case since the material was fully cured. Hardener leaching was not present, as supported by ATR-FT-IR. If the hardener washout or residual crosslinking was the case, it would show a decrease in peak intensity at wavenumber corresponding to unreacted amine groups at 3200–3500 cm^−1^ [43], which was not observed in the ATR-FT-IR spectra. It should also be noted that any changes to the epoxy material below IR sensitivity cannot be detected.

Whilst it was found that leaching of hardener is improbable, leaching of chemicals that are initially present in epoxy resin, such as epichlorohydrin, was found likely, using a combination of EDX, HR-ICP-MS, and FT-NIR. Based on the HR-ICP-MS data and FT-NIR intensity of the corresponding peak at 4535 cm^−1^, the leached amount of epichlorohydrin was estimated to be 75 μg/g of resin, and the initial concentration of epichlorohydrin in the resin was estimated to be 137 μg/g.

HR-ICP-MS showed leaching of Cl-containing compounds or chloride ions. It should be noted that the DGEBA epoxy component itself, is a product of O-alkylation of Bisphenol A and epichlorohydrin. Upon such reaction, HCl is released. However, the reaction is conducted in the presence of sodium hydroxide NaOH, meaning that HCl is being neutralized [44]. It is possible that some of the Cl compounds, i.e., NaCl, unneutralized HCl or unreacted epichlorohydrin, were present in the initial dry resin. It is likely that these compounds were washed out from the epoxy system during hygrothermal aging. Furthermore, the pH measurements (Figure 7) indicate a release of acidic compounds from the resin, i.e., HCl.

To sum up, the leaching of hardener was not observed, whilst the leaching of epoxy compounds, such as epichlorohydrin, and leaching of impurities, such as HCl and NaCl, was present.

### 5.3. Thermo-Oxidation

Thermo-oxidation is highly selective [45], and the main source of radicals is the decomposition of the most thermally unstable chemical species [26]. Each carbon atom in α position of an electronegative atom, such as O and N, has a decreased dissociation energy and increased reactivity in the radical chain propagation, thus resulting in “weak points” in the network structure [4,28].

Increase in the intensity of the carbonyl group peak (νC=O; 1730–1740 cm^−1^) [4,8,10,28,39,40], for aged epoxy, is related to an oxidation process taking place. Carbonyl formation can result from various pathways [28]. Thermo-oxidation involving chain scission and appearance of double bonds; carbonyl and amide species have been reported in DGEBA/IPDA and DGEBA/POPA systems [4,21,41]. Appearance of diphenylketones was reported in another work [46]. In this work, the appearance of amide species (C=O; 1644 cm^−1^ and N-H; 3325 cm^−1^) [8,43] and diphenylketones (C=O; 1660 cm^−1^) [46] due to thermo-oxidation was not observed. Double bond formation was not observed, as indicated by C=C bands close to 1600 cm^−1^ (1624 cm^−1^ and 1593 cm^−1^) [28].

**Figure 10:**
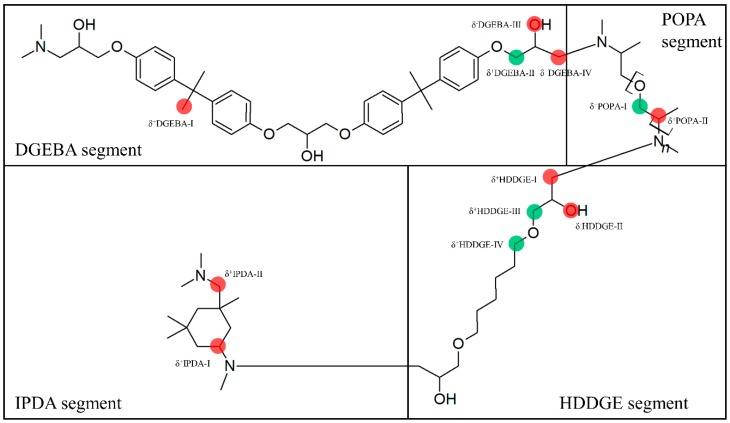
Chemical structure of the studied DGEBA/HDDGE/IPDA/POPA amine epoxy network (mixing ratios are not considered). Marked sites represent “weak points” for radical attack in the network. Sites marked in red are excluded based on experimental evidence and literature. Sites marked in green are the main reactive sites.

Chemical structure of a cured amine epoxy network (not considering the mixing proportions), is shown in Figure 10. “Weak points” for radical attack in the network were identified and are shown in Figure 10. In the network, 12 unique sites potentially involved in thermo-oxidation were found. Furthermore, 8 of these sites were excluded based on experimental evidence and literature. Sites marked in green were the identified main reactive sites (δ^+^DGEBA-II, δ^+^POPA-I, δ^+^HDDGE-III, and δ^+^HDDGE-IV).

**Scheme 1:**
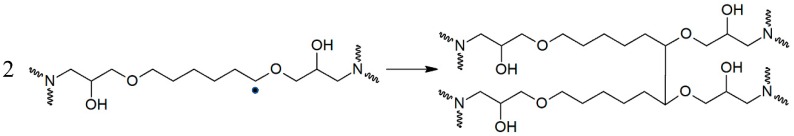
Crosslinking reaction of the HDDGE segments via reactive sites δ^+^HDDGE-IV.

*T*_g_ can be regarded as a useful parameter revealing chemical changes for polymers [47]. The *T*_g_ of a redried epoxy (84.7 °C) was slightly higher than for the initial material (81.7 °C), indicating that no chain scission occurred [7,41]. A likely reason of a *T*_g_ increase was a combination of polymer relaxation [13], anti-plasticizing effect of leaching, and a minor crosslinking [7,27] of the HDDGE segments (site δ^+^HDDGE-IV). The thermo-oxidative crosslinking mechanism has been reported for an amine epoxy with a curing agent DGEBU similar to HDDGE in another work [27]. An analogous crosslinking mechanism of HDDGE segments is proposed in Scheme 1, involving sites δ^+^HDDGE-IV.

The only way to form amides is an oxidation of amino methylenes near to network nodes [28]. There was no increase in intensity of the bands corresponding to amide species (C=O; 1644 cm^−1^ and N-H; 3325 cm^−1^) [8,43]. This excluded the following “weak points” as potential reactive sites: δ^+^DGEBA-IV, δ^+^POPA-II, δ^+^HDDGE-I, δ^+^IPDA-I, and δ^+^IPDA-II. Moreover, the formation of amide species is linked to chain scission, which was not present, as indicated by *T*_g_ measurements.

The linkage between the aromatic rings may also be sensitive to oxidation [10,25]. Carbonyl formation via acetophenone groups due to radical attack on δ^+^DGEBA-I site was not observed, as indicated by the absence of the increase of the acetophenone “in-chain” group band (1684 cm^−1^) [10] and diphenylketones (C=O; 1660 cm^−1^) [46].

The formation of carbonyl groups may result from oxidation of the secondary alcohol groups in cured resin [28,46]. This oxidation process is accompanied by the decrease of the band at 1237 cm^−1^, representing the characteristic C-O band in secondary alcohol groups [46]. No changes to this band were observed, which excluded the following “weak points” as potential reactive sites: δ^−^DGEBA-III and δ^−^HDDGE-II. Furthermore, in the hydroxyl domain (≈3800–2500 cm^−1^) [10] no changes were observed, which further supported this conclusion.

Noteworthy that all three reactive sites (δ^+^DGEBA-II, δ^+^POPA-I, and δ^+^HDDGE-III) involved in carbonyl formation have similar structures. Whilst δ^+^POPA-I contains the polyoxypropylene moiety, the other two sites (δ^+^DGEBA-II and δ^+^HDDGE-III) are identical and contain the *i*-propanol moiety.

**Scheme 2:**
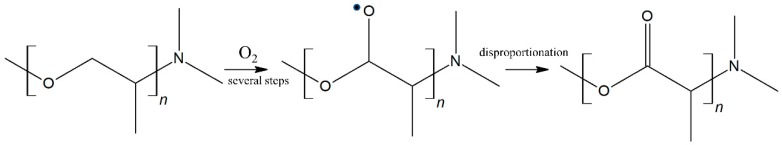
Carbonyl formation involving polyoxypropylene moiety on reactive site δ^+^POPA-I.

δ^+^POPA-I site as a polyoxypropylene moiety-containing segment is very susceptible to radical attack under oxidation, due to low stability of the tertiary C-H bond and the destabilizing effect of the neighboring ether group [10,41]. The proposed carbonyl formation reaction on this site is shown in Scheme 2.

**Scheme 3:**
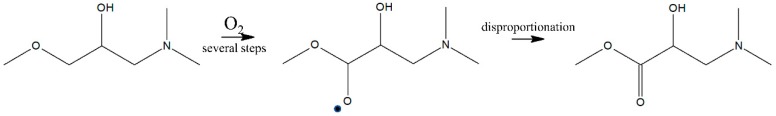
Carbonyl formation reaction involving *i*-propanol moiety on reactive sites δ^+^DGEBA-II and δ^+^HDDGE-III.

The δ^+^DGEBA-II and δ^+^HDDGE-III sites as *i*-propanol moiety-containing segments are also highly susceptible to oxidation [4,28]. The carbonyl formation on these two sites follows the same reaction, as shown in Scheme 3 [28].

Contrary to the DGEBA/IPDA and DGEBA/POPA binary amine epoxies [4,21,42], the results suggest that a combination of DGEBA/HDDGE/IPDA/POPA stops the oxidative chain scission and amide formation. It is not clear what exactly causes such change, but it is possible that some of the identified reactive sites operate as weak “sacrificial” centers, as suggested in another work [17], thus protecting the remaining structure.

To sum up, the results indicated that there was no chain scission, double bonds and amide groups were not formed, whilst the evolution of carbonyl groups in the macromolecular backbone and minor crosslinking of HDDGE segments occurred.

### 5.4. The Cause of Yellowing

The change in color is irreversible, and is related to the irreversible aging mechanism. Two irreversible phenomena were identified: (1) Leaching of an epoxy compound epichlorohydrin and impurities, i.e., HCl and NaCl; (2) Formation of carbonyl groups C=O in the polymeric backbone due to thermo-oxidation (sites δ^+^DGEBA-II, δ^+^POPA-I, and δ^+^HDDGE-III, shown in Figure 10). Both impurities and epichlorohydrin are colorless and do not cause discoloration. The oxidative evolution of carbonyl groups in the resin was the reason for the yellowing. It has been reported for other polymers that carbonyl formation can cause a change in color [48,49]. Furthermore, the yellowing phenomenon of a polyurethane resin was found to be linked to the mechanism of carbonyl formation in the macromolecular backbone, caused by oxidation [49].

### 5.5. Increasing Epoxy Service Life

Yellowing of the studied epoxy was linked to the formation of carbonyl groups in the macromolecular backbone. Based on other studies [25,50,51], conclusion can be drawn that carbonyl formation can be slowed down by using phenolic antioxidants, such as hindered phenols, which are used as stabilizers for various plastics and rubbers [50,51]. These compounds act as radical scavengers and can prevent thermo-oxidative yellowing. An effective way of introducing stabilization for epoxies, when mechanical properties are not concerned, is a co-curing procedure of epoxy with resole, which has hindered phenol moieties [50,51].

### 5.6. On the Similarity of Yellowing and Thermo-Oxidation Kinetics

Kinetics of epoxy yellowing and thermo-oxidative carbonyl formation found in literature, undoubtedly show common trends [6,25,28,41].

Yellowing of epoxies is not linear in time and is characterized by three stages [6]: (1) The induction period with little or no yellowing [6]; oxidation kinetics also display an induction period with a strongly auto-accelerated character [28]; (2) The steady state period, during which yellowing is high and constant [6]; (3) The declining rate period, during which the increase in yellowing is occurring at a slower rate [6], which is in agreement with another study on thermo-oxidation, stating that high oxidation levels can result in reduced sensitivity to further oxidation [25]. In the long term, in some cases, there is a horizontal asymptote in carbonyl formation kinetics, indicating equilibrium or a saturation phase, but it is not systematically observed [41]. The kinetics have a strong dependence on temperature and presence of antioxidants [28]; the kinetics follow the Arrhenius principle over a wide temperature range [25,26].

## 6. Conclusions

This work discussed the mechanism of yellowing of a DGEBA/HDDGE/IPDA/POPA amine epoxy. Based on the results of FT-NIR, ATR-FT-IR, EDX, HR-ICP-MS, pH measurements, optical microscopy, SEM, and DMTA, the following conclusions have been made:

1. Yellowing occurred due to the thermo-oxidative carbonyl formation in the epoxy carbon-carbon backbone via nucleophilic radical attack. The change in color was irreversible. Morphology was found to be unaffected.
2. No chain scission (hydrolysis or oxidation-induced) was present, whilst thermo-oxidation and leaching occurred.
3. Compounds involved in leaching were identified to be epichlorohydrin and inorganic impurities but were unrelated to yellowing.
4. Four unique reactive sites responsible for thermo-oxidation were found. One reactive site was involved in minor thermo-oxidative crosslinking of the HDDGE segments, while three other sites were linked to carbonyl formation. Noteworthy that all three sites involved in carbonyl formation had similar structures, containing highly reactive polyoxypropylene and *i*-propanol moieties. Respective reactions were proposed.
5. It is speculated that yellowing could be prevented or delayed by adding phenolic antioxidants, such as hindered phenols.
